# Utilization of medical rehabilitation services among older Poles: results of the PolSenior study

**DOI:** 10.1007/s41999-018-0077-8

**Published:** 2018-06-20

**Authors:** Aleksandra Szybalska, Katarzyna Broczek, Przemysław Slusarczyk, Ewa Kozdron, Jerzy Chudek, Monika Puzianowska-Kuznicka, Tomasz Kostka, Anna Skalska, Malgorzata Mossakowska

**Affiliations:** 1 International Institute of Molecular and Cell Biology in Warsaw, 4 Ks. Trojdena Street, 02-109 Warsaw, Poland; 2 Department of Geriatrics, Medical University of Warsaw, Warsaw, Poland; 3 Department of Recreation Methodology, Faculty of Tourism and Recreation, Jozef Pilsudski University of Physical Education in Warsaw, Warsaw, Poland; 4 Department of Internal Medicine and Oncological Chemotherapy, Medical Faculty in Katowice, Medical University of Silesia, Katowice, Poland; 5 Department of Human Epigenetics, Mossakowski Medical Research Centre, PAS, Warsaw, Poland; 6 Department of Geriatrics and Gerontology, Medical Centre of Postgraduate Education, Warsaw, Poland; 7 Department of Geriatrics, Medical University of Lodz, Lodz, Poland; 8 Department of Internal Medicine and Gerontology, Jagiellonian University Medical College, Cracow, Poland

**Keywords:** Rehabilitation medicine, Aging, Population-based study, Disability, PolSenior study, Health inequalities

## Abstract

**Background:**

Rehabilitation tailored to older adults’ needs might improve their functional performance and quality of life, as well as increase social participation. The aim of the study was to evaluate the use of medical rehabilitation services among older Poles in relation to socio-economic and health-related determinants.

**Materials and methods:**

Data regarding medical rehabilitation were obtained from the nationwide, multidisciplinary PolSenior project (2007–2012) conducted on representative sample of 4813 respondents (48.3% women) aged 65+ years. Socio-economic status, physical functioning, falls, chronic pain, and formal disability occurrence, as well as self-rated health were accounted for.

**Results:**

One in six respondents (18.9% women vs. 15.8% men, *p* < 0.005) underwent medical rehabilitation during 12 months prior to the survey. Respondents mostly received electrotherapy or light radiation therapy (61.3%). Multivariate logistic regression analysis revealed that women aged 80+ years and men aged 90+ years had a significantly lower chance of using rehabilitation services compared to the youngest study participants (65–69 y.o.). City dwellers used rehabilitation services nearly twice as frequently as rural dwellers. Respondents with university education level were most likely to take part in these services. Dependence in IADL decreased participation in medical rehabilitation, while formal disability and chronic pain promoted utilization of rehabilitation services.

**Conclusions:**

Younger age, city dwelling, higher education, functional independence, formal disability certificate, and chronic pain increased participation in medical rehabilitation. Such results of the study should be considered in planning actions towards reducing health inequalities at the national level and promoting health and well-being among older adults.

## Introduction

Aging of developed societies is a burden for health care systems, including rehabilitation services designed to optimize functioning and reduce disability in individuals with health conditions in interaction with their environment [1].

Effective rehabilitation and reablement are mentioned as one of ten components of care for older adults [2, 3]. Preventive or treatment rehabilitation services should be complex, adequate, and well-tailored to the needs and expectations of older people. Researchers emphasize the importance of a multidisciplinary team of professionals, as well as patients’ and their family members’ engagement in rehabilitation process for its effectiveness [2, 4, 5]. Patients as well as medical professionals see the need for patient-centered goal setting; however, implementing such interventions is perceived as challenging in geriatric rehabilitation [6].

Rehabilitation services for prevention of frailty and disability are of special importance for public health in aging societies. Positive outcomes of rehabilitation on older individuals’ activities of daily living and quality of life have been shown in different settings and cultural backgrounds [7–9]. Early physical rehabilitation during hospitalization led to shorter stay in acute geriatric wards, as well as reduced readmission rate in orthopedic surgery departments [10, 11].

Numerous authors underline the importance of inequalities in health particularly among older adults, both in macro and micro scale [12–15]. In Poland, inequalities in health might be increased by low expenditure for health care per capita and shortage of medical staff compared to other countries [16]. In the newest Euro Health Consumer Index Report, comparing the European health care systems performance, Poland was rated 29th among 35 countries [17].

Medical rehabilitation services in Poland are financed from public and private resources. Insurance-covered services are provided by the National Health Fund and the State Fund for Rehabilitation of Disabled Persons. However, this system is not sufficient and, due to long waiting lists, a significant number of patients utilize commercial rehabilitation services.

The purpose of the study was to evaluate utilization of medical rehabilitation, its socio-economic correlates, and potential determinants of inequality in health of Polish older adult population.

## Materials and methods

Data regarding medical rehabilitation services were obtained from the nationwide, multidisciplinary PolSenior project titled “Medical, psychological and socioeconomic aspects of aging in Poland” conducted from 2007 to 2012. Study cohort, representative for the Polish population aged 65 years and over, comprised 4979 participants. The representativeness of the sample recruited using three stage stratified, proportional draw, was obtained by weighting for demographic structure of the older Polish population. The fieldwork was conducted in respondents’ homes by trained nurses. Ethical approval (no. KNW-6501-38/I//08) was obtained from the Bioethics Commission of the Medical University of Silesia in Katowice. All study participants or their proxies signed informed consent forms. The design of the PolSenior project was described previously [18].

Data regarding utilization of medical rehabilitation within 12 months prior to the survey and types of services were drawn from the socio-economic questionnaire (available on-line: http://polsenior.iimcb.gov.pl/en/questionnaire). Sources of financing of medical rehabilitation services (insurance-covered vs. private resources) were not analyzed. Socio-demographic variables included in the analyses were: age, place of residence, education level, type of work before retirement, self-assessed economic status, and having formal disability certificate (classified to three degrees: minor, moderate, severe). Other analyzed variables were: functional status, chronic pain occurrence, and its intensity, falls within 12 months prior to the survey and self-rated health (SRH).

Functional status was evaluated using two tools: the Katz Index of Activities of Daily Living Scale (ADL) and the Lawton Instrumental Activities of Daily Living Scale (IADL) [19, 20]. Based on the ADL score, participants were classified as: dependent (0–2 points), partially dependent (3–4 points), and independent (5–6 points). According to the IADL score, respondents were classified as: dependent, partially dependent, and independent (8–18 points, 19–23 points, and 24 points, respectively). Detailed analyses of prevalence of falls, as well as prevalence of chronic pain in the PolSenior study group were described previously [21, 22].

Self-rated health was measured using 0–10 pt visual analog scale [18] and scored as poor (0–3 pts), fair (4–6 pts), and good (7–10 pts) health.

The PolSenior project questionnaires included two types of questions: regarding facts (addressed to respondents and/or their proxies) and regarding opinions (addressed only to respondents) [18]. In the current study, among aforementioned variables regarding respondents’ opinions were: self-assessed economic status, pain intensity, and SRH.

### Data analysis

Statistical analyses were performed using Statistica 10.0 software (StatSoft, Tulsa, OK, USA). The *χ*^2^ and Cochran-Armitage for trend tests were performed to identify factors related to utilization of medical rehabilitation in the study group.

Variables significantly associated with rehabilitation use were subsequently included into a multivariate logistic regression model. In the model, IADL was chosen as determinant of functional status, and, consequently, ADL was excluded as dependent variable. Data were presented as odds ratios (ORs) with 95% confidence intervals (95%CIs).

Presented analyses differed in terms of the number of observations because of some missing data.

In all analyses, *p* value < 0.05 was considered as statistically significant.

## Results

Data regarding medical rehabilitation were collected from 4813 participants, constituting 96.7% of the PolSenior study cohort aged 65 years and over. One in six respondents (17.3%), 18.9% women and 15.8% men (*p* < 0.005), took part in medical rehabilitation during 12 months prior to the study. After weighting for demographic structure of the older Polish population, the utilization of rehabilitation services was higher than in the PolSenior study group (22.3 vs. 17.3%), with greater difference between women and men (24.7 vs. 18.5%; *p* < 0.001).

**Fig. 1:**
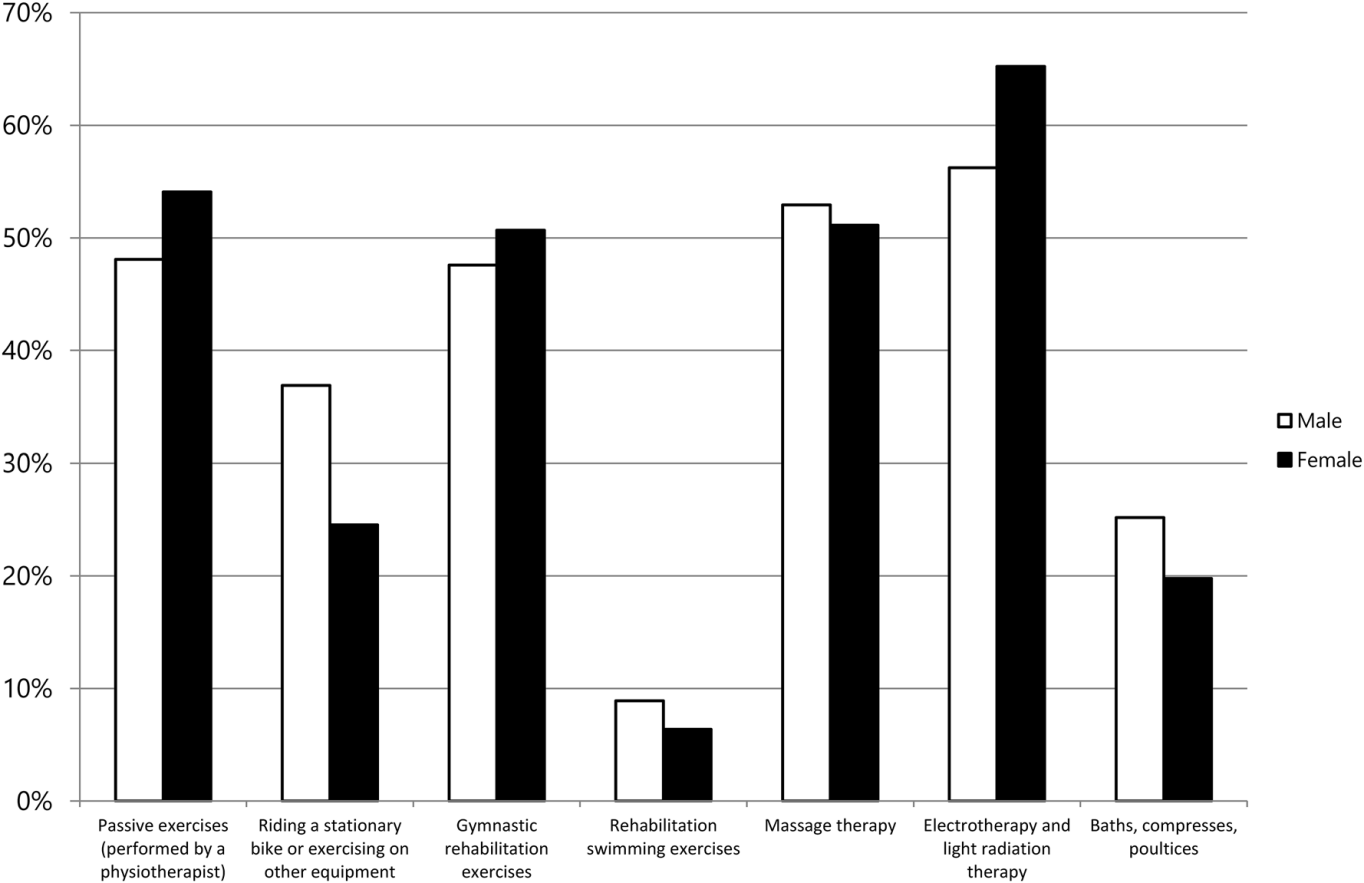
The utilization of medical rehabilitation services in terms of gender

Rehabilitation services utilized most frequently by the PolSenior study respondents were: electrotherapy and light radiation therapy (61.3%), massage therapy (52.0%), or passive exercises (51.4%). Distribution of the type of medical rehabilitation services according to gender is presented in Fig. 1.

The utilization of rehabilitation services decreased with age. Participation in medical rehabilitation was declared by every fourth respondent in the youngest age cohort (65–69 y.o.), every fifth aged 75–79 years, and every sixteenth aged 90 years and over (*p* < 0.001). In younger age cohorts (up to 84 y.o.) women, while in older age cohorts (85 y.o. and over) men, dominated as beneficiaries of the services—*p* < 0.001 (Table 1). City dwellers (particularly living in the largest cities) underwent medical rehabilitation more than twice as often as village dwellers (22.6 vs. 9.4%; *p* < 0.001). Respondents with university education level were more than 2.5 times as often as those with primary education among rehabilitation users (31.2 vs. 11.8%; *p* < 0.001) and, respectively, more than 5 times as often as those without formal education (31.2 vs. 5.8%; *p* < 0.001). Notably, respondents with vocational education were the second subgroup after those with higher education (29.1 vs. 31.2%, respectively), which used the rehabilitation services most frequently.

**Table 1:** Characteristics of the study group according to utilization of medical rehabilitation

Variable	Characteristics	Women			Men			Total		
		*N*	Use of rehabilitation services [*n*; %]	*p*	*N*	Use of rehabilitation services [*n*; %]	*p*	*N*	Use of rehabilitation services [*n*; %]	*p*
Age [years]	65–69	400	121; 30.3	**< 0.001**	368	72; 19.6	**< 0.001**	768	193; 25.1	**< 0.001**
	70–74	431	123; 28.5		470	88; 18.7		901	211; 23.4	
	75–79	388	85; 21.9		426	85; 20.0		814	170; 20.9	
	80–84	353	59; 16.7		408	60; 14.7		761	119; 15.6	
	85–89	378	35; 9.3		457	58; 12.7		835	93; 11.1	
	90 +	375	17; 4.5		359	30; 8.4		734	47; 6.4	
Place of residence	Rural area	967	103; 10.7	**< 0.001**	970	79; 8.1	**< 0.001**	1937	182; 9.4	**< 0.001**
	Urban area	1358	337; 24.8		1518	314; 20.7		2876	651; 22.6	
Place of residence [number of residents]	Rural area	967	103; 10.7	**< 0.001**	970	79; 8.1	**< 0.001**	1937	182; 9.4	**< 0.001**
	City ≤ 20,000	311	64; 20.6		332	53; 16.0		643	117; 18.2	
	City > 20,000-50,000	275	61; 22.2		312	61; 19.6		587	122; 20.8	
	City > 50,000-200,000	280	70; 25.0		338	78; 23.1		618	148; 23.9	
	City > 200,000-500,000	105	27; 25.7		116	23; 19.8		221	50; 22.6	
	City > 500,000	387	115; 29.7		420	99; 23.6		807	214; 26.5	
Education	Lack of education	401	25; 6.2	**< 0.001**	272	14; 5.1	**< 0.001**	673	39; 5.8	**< 0.001**
	Primary	1149	157; 13.7		971	92; 9.5		2120	249; 11.8	
	Vocational	423	148; 35.0		496	119; 24.0		919	267; 29.1	
	Secondary	163	43; 26.4		435	81; 18.6		598	124; 20.7	
	Bachelor	104	36; 34.6		177	41; 23.2		281	77; 27.4	
	MCs^a^	75	30; 40.0		132	45; 34.1		207	75; 36.2	
Type of work	Blue-collar worker	1005	160; 15.9	**< 0.001**	1407	182; 12.9	**< 0.001**	2412	342; 14.2	**< 0.001**
	Farmer	372	29; 7.8		253	16; 6.3		625	45; 7.2	
	White-collar worker	555	193; 34.8		641	159; 24.8		1196	352; 29.4	
	Other^b^	143	36; 25.2		155	33; 21.3		298	69; 23.2	
Self-reported economic status	Enough money for all needs	1291	281; 21.8	NS	1682	283; 16.8	NS	2973	564; 19.0	NS
	Enough money to make a living, but not for all needs	643	123; 19.1		511	86; 16.8		1154	209; 18.1	
	Not enough money	113	16; 14.2		67	6; 9.0		180	22; 12.2	
Disability certificate	No	1657	260; 15.7	**< 0.001**	1705	208; 12.2	**<0.001**	3362	468; 13.9	**<0.001**
	Yes	623	176; 28.3		739	181; 24.5		1362	357; 26.2	
Disability degree	Minor	95	38; 40.0	**< 0.001**	109	26; 23.9	NS	204	64; 31.4	**< 0.001**
	Moderate	212	79; 37.3		217	61; 28.1		429	140; 32.6	
	Severe	291	54; 18.6		385	91; 23.6		676	145; 21.5	
^c^ADL status	Independent	1980	404; 20.4	**< 0.001**	2190	356; 16.3	NS	4170	760; 18.2	**< 0.001**
	Partially dependent	152	15; 9.9		145	15; 10.3		297	30; 10.1	
	Dependent	163	15; 9.2		124	18; 14.5		287	33; 11.5	
^d^IADL status	Independent	1047	298; 28.5	**< 0.001**	1222	236; 19.3	**< 0.001**	2269	534; 23.5	**< 0.001**
	Partially dependent	545	81; 14.9		549	91; 16.6		1094	172; 15.7	
	Dependent	722	59; 8.2		702	66; 9.4		1424	125; 8.8	
Chronic pain	No	1185	174; 14.7	**< 0.001**	1594	209; 13.1	**< 0.001**	2779	383; 13.8	**< 0.001**
	Yes	1125	263; 23.4		875	180; 20.6		2000	443; 22.2	
Chronic pain intensity	Mild	178	35; 19.7	NS	204	54; 26.5	**0.013**	382	89; 23.3	NS
	Moderate	493	121; 24.5		424	73; 17.2		917	194; 21.2	
	Severe	390	100; 25.6		204	50; 24.5		594	150; 25.3	
Falls	No	1691	334; 19.8	NS	1980	316; 16.0	NS	3671	650; 17.7	NS
	Yes	608	103; 16.9		479	72; 15.0		1087	175; 16.1	
^e^SRH	Poor	226	32; 14.2	**0.015**	199	27; 13.6	NS	425	59; 13.9	**0.003**
	Fair	1133	249; 22.0		1135	202; 17.8		2268	451; 19.9	
	Good	759	142; 18.7		986	153; 15.5		1745	295; 16.9	

p value <0.05 is statistically significant

*N* total number of participants in age groups, *n*: number of rehabilitation users in age groups

^a^*MCs* master’s degree

^b^Other worker including salespersons, owners of a trade or service workshop, small entrepreneurs, uniformed services officers

^c^*ADL* activities of daily living

^d^*IADL* instrumental activities of daily living

^e^*SRH* self-rated health

Among white-collar workers, the utilization was nearly 2 times higher than in blue-collar workers and nearly 4 times higher than among farmers (29.4 vs. 14.2 vs. 7.2%, respectively; *p* < 0.001).

There was no association between the self-assessed economic status and use of rehabilitation services.

Disability certificate holders used rehabilitation services nearly 2 times more often than those without formal disability status (26.2 vs. 13.9%; *p* < 0.001). Respondents with formal status of moderate disability were most frequent rehabilitation users (32.6 vs. 31.4% in those with minor and 21.5% in those with severe disability; *p* < 0.001).

In addition, respondents independent in IADL utilized rehabilitation services over 2.5 times more often than dependent ones (23.5 vs. 8.8%; *p* < 0.001). This proportion was less visible for independent vs. dependent in ADL, 1.5 times difference (18.2 vs. 11.5%; *p* < 0.001). In the last case, the difference among men was not statistically significant.

Respondents who have reported chronic pain were more likely to use rehabilitation services (22.2 vs. 13.8%; *p* < 0.001). However, association between intensity of chronic pain and utilization of rehabilitation services was statistically significant only in men.

Respondents who reported fair SRH participated in rehabilitation most frequently in the whole group (19.9% for fair vs. 16.9% for good and 13.9% for poor SRH; *p* = 0.03), and among women (Table 1). The analyses revealed no association between the prevalence of falls and the use of rehabilitation services.

Multivariate logistic regression analysis indicated that women aged 80 years and over had a lower chance of taking part in rehabilitation than the youngest women study participants (65–69 y.o.). In men, a significant difference was noted only between the youngest and the oldest (90 y.o. and over) male cohort. City dwellers were more likely to use rehabilitation services compared to village inhabitants. University level education promoted participation in rehabilitation in both genders. Dependence in IADL was inversely associated with utilization of the analyzed services among women and men. Disability certificate holders and those who reported chronic pain were more likely to take part in medical rehabilitation (Table 2).

**Table 2:** Factors associated with the utilization of medical rehabilitation among the PolSenior study respondents. Results of the multivariate logistic regression analysis

Variable	Characteristics	Women			Men		
		OR	95% CI	*p* value	OR	95% CI	*p* value
Age [years]	65–69	Ref.			Ref.		
	70–74	0.89	0.64–1.24	NS	0.94	0.65–1.36	NS
	75–79	0.76	0.53–1.09	NS	1.10	0.76–1.61	NS
	80–84	0.66	0.44–0.99	**0.046**	0.74	0.49–1.12	NS
	85–89	0.36	0.21–0.60	**<0.001**	0.72	0.47–1.10	NS
	90+	0.26	0.14–0.48	**<0.001**	0.50	0.30–0.86	**0.012**
Place of residence	Rural area	Ref.			Ref.		
	Urban area	1.91	1.45–2.52	**<0.001**	1.93	1.43–2.61	**<0.001**
Education level	Primary/lack of education	ref.			ref.		
	Vocational	1.64	1.07–2.51	**0.024**	1.72	1.22–2.43	**0.002**
	Secondary	2.48	1.85–3.32	**<0.001**	2.19	1.58–3.02	**<0.001**
	Bachelor	1.90	1.17–3.10	**0.009**	2.05	1.31–3.19	**0.002**
	MCs^a^	2.82	1.63–4.88	**<0.001**	3.84	2.42–6.07	**<0.001**
^b^IADL status	Independent	Ref.			Ref.		
	Partially dependent	0.57	0.42–0.79	**0.001**	1.03	0.76–1.40	NS
	Dependent	0.61	0.40–0.92	**0.018**	0.67	0.47–0.97	**0.035**
Disability certificate	No	ref.			ref.		
	Yes	1.93	1.50–2.47	**<0.001**	2.29	1.79–2.93	**<0.001**
Chronic pain	No	Ref.			ref.		
	Yes	2.03	1.59–2.60	**<0.001**	1.79	1.40–2.27	**<0.001**
^c^SRH	Good	ref.			ref.		
	Fair	1.21	0.93–1.57	NS	1.15	0.89–1.47	NS
	Poor	0.86	0.53–1.38	NS	0.78	0.48–1.29	NS

p value <0.05 is statistically significant

^a^*MCs* master’s degree

^b^*IADL* instrumental activities of daily living

^c^*SRH* self-rated health

## Discussion

The present study describes the socio-economic and health-related determinants of rehabilitation use among older Poles and characterizes the inequalities in the access to these services. Responding adequately to the demographic and epidemiological trends and tailoring the health care systems including rehabilitation to the patients’ needs has been reflected in recommendations and good practices formulated by international bodies [1, 23–26]. The PolSenior project might contribute to implementation of such recommendations at the national level through extending knowledge on utilization of health care services in a representative sample of older adults.

In our study, one in six respondents participated in medical rehabilitation during 12 months prior to the survey. Electrotherapy and light radiation therapy, massage therapy, or passive exercises were among the most commonly used services. Beneficial effects of these therapies on age-related health problems have been previously demonstrated [27–30].

Results of the PolSenior study showed that the use of rehabilitation services decreased with age, from one in four in the youngest to one in sixteen in the oldest age cohort. Notwithstanding, published data indicate that rehabilitation might be useful and cost-effective in advanced age as shown by Davis et al. [31] regarding falls prevention among community dwellers aged 80 years and over.

In the current study, women participated in medical rehabilitation more frequently than men (18.9 vs. 15.8%; *p* < 0.005). Notably, when separate age cohorts were analyzed, the opposite was noticed in the oldest old, above the age of 85 years (Table 1). Different patterns in rehabilitation services use might be related to differences in health status between women and men and gender-based health inequalities. Older women have greater demand for medical services due to worse functional status and higher multimorbidity rate than men but, concomitantly, they are at increased risk of being exposed to limitations in access to the health care systems related to long-term disability as shown for the European region [13, 32]. Comorbidity increases the need for rehabilitation, but, at the same time, it augments the risk of intercurrent diseases during recovery, as shown by Kabboord et al. [33] in patients undergoing geriatric stroke rehabilitation. Relationship between comorbidity and disability is complex, as shown in ilSIRENTE Study (Ageing and Longevity Study in the Sirente geographic area) where functional performance more strongly correlated with 4-year all-cause mortality than multimorbidity among frail individuals [34].

In addition to advanced age and functional dependence, rural dwelling and lower education level were among factors reducing utilization of medical rehabilitation by Polish seniors. Similarly to our findings, better educated participants of the international Survey of Health, Ageing and Retirement (SHARE) aged 50 years and over, were more likely to contact the specialist, visit the dentist or be hospitalized in comparison to less educated ones [35]. Rural dwelling is related to limited access to medical services including rehabilitation, particularly for older adults with impaired mobility [36].

Surprisingly, we did not observe any relationships between the self-rated economic status and the utilization of rehabilitation services. However, objective measures of the incomes were not included in the analysis because of high rate of refusal to reveal financial data. A study carried out in USA showed positive correlation of personal income and massage therapy use among adults aged 60 years and over [37]. Based on national health surveys data from 18 countries within the Organisation for Economic Cooperation and Development (OECD), it was shown that inhabitants with higher income were more likely to contact a doctor or utilize dental or preventive care than the less wealthy. Poland was among countries with significant income-related inequalities in primary care, specialist and dentist visits [38].

In the current study, respondents holding a formal disability certificate had higher participation in medical rehabilitation independently of functional performance. We speculate that formal disability holders acquired experience with certain bureaucracy, and this might have facilitated their access to limited services in an inefficient health care system [17]. Previous analyses within the PolSenior study revealed that respondents with chronic pain contacted family doctors more often than those without chronic pain [22]. The same pattern was observed in the present study; women and men reporting chronic pain had a nearly twice higher chance for utilization of rehabilitation services in comparison to those without pain.

Our study indicated no association between SRH and participation in medical rehabilitation, whereas Figueiredo et al. [39] found SRH to be a useful tool for evaluating medical service needs among older adults discharged from the hospital. Potential reason for the aforementioned difference could be the methodology of presented surveys since the PolSenior study group comprised of community dwellers.

The strength of the present study was providing data for a large group of older community dwellers with high proportion of men, as well as very old individuals who are underrepresented in most gerontological studies. Weights’ adjustment for demographic structure of the older Polish population enabled generalization of the obtained results to the nationwide perspective. The authors perceive that the limitation of this study is lack of data concerning resources of financing of medical rehabilitation services, as well as unknown reasons for rehabilitation (preventive or therapeutic) in the PolSenior respondents.

In conclusion, determinants related to the utilization of medical rehabilitation services were: younger age, city dwelling, higher education, independence in IADL, formal disability, as well as chronic pain. Healthy and active aging, as well as reducing inequalities is emphasized as a priority of the European policy framework Health 2020 [15, 40]. Therefore, findings of the present study might be useful for policy makers in distributing health care system resources especially in the area of rehabilitation medicine in the rapidly aging European region. Further research of health services use by older adults will be important as geriatric medicine and rehabilitation are perceived as the key health strategy for the 21st century [23].
